# Splenic Infarction: An Unusual Complication of Salmonella Infection

**DOI:** 10.7759/cureus.94171

**Published:** 2025-10-09

**Authors:** Vinod Kumar, Amtoj Singh Lamba, Binita Jora, Monica Gupta, Harsimran Kaur

**Affiliations:** 1 General Medicine, Government Medical College and Hospital, Chandigarh, IND; 2 Internal Medicine, Government Medical College and Hospital, Chandigarh, IND; 3 Radiodiagnosis, Government Medical College and Hospital, Chandigarh, IND

**Keywords:** enteric fever (typhoid fever), immunocompetent adult, salmonella paratyphi a, salmonella typhi, splenic infarction

## Abstract

Splenic infarction is an uncommon diagnosis, often overlooked due to its non-specific clinical presentation. While a splenic abscess is common, infarction due to infective processes is rare. Among infections, *Salmonella *is infrequently implicated, and splenic infarct secondary to typhoid or paratyphoid fever is exceptionally rare. We report the case of a young adult man who presented with acute fever and pain in the abdomen and was subsequently diagnosed with splenic infarction. A meticulous diagnostic workup excluded common causes such as infective endocarditis, hematological malignancies, hypercoagulable states, and sickle cell disorder, ultimately identifying non-lactose fermenting colonies positive for *Salmonella paratyphi A* as the etiology. Recognition of such atypical abdominal complications of typhoid is essential in endemic areas to prevent missed diagnosis and optimize management.

## Introduction

In developing nations, *Salmonella *infections are prevalent and largely attributable to inadequate sanitation, limited access to safe drinking water, and insufficient training among food handlers. The predominant causative agent of enteric fever is *Salmonella enterica serovar Typhi.* However, other serovars, particularly Paratyphi A, B, and C, can induce a clinically indistinguishable illness. Humans serve as the exclusive reservoir for both. The term enteric fever encompasses both typhoid and paratyphoid fevers, and these terms are frequently used interchangeably in clinical practice [1,2].

Typhoid fever can present with a plethora of non-specific symptoms, such as fever, nausea, vomiting, abdominal pain, and may be accompanied by a range of complications [3]. Most patients with *Salmonella* infection present with an acute gastroenteritis-like illness that is typically self-resolving. However, in some instances, the infection may progress to bacteremia or result in localized extra-intestinal complications [4]. Gastrointestinal involvement in enteric fever may manifest clinically as jaundice, hepatosplenomegaly, acalculous cholecystitis, intestinal perforation, or hemorrhage. A smaller subset of patients can develop transaminitis, cholestatic hepatitis, serositis, or peritonitis [5]. Reports of *Salmonella*-associated isolated splenic infarction remain exceedingly uncommon, particularly that caused by *Salmonella enterica serovar Paratyphi A*. We describe an unusual case of splenic infarction occurring in the setting of paratyphoid fever.

## Case presentation

A 34-year-old man was admitted to the emergency department with an acute onset of fever and abdominal pain lasting four days. The fever, documented up to 101°F, was associated with chills and rigors and subsided temporarily with antipyretics. The abdominal pain was insidious in onset, dull aching in nature, and predominantly localized to the left upper quadrant. There were no associated symptoms such as nausea, vomiting, jaundice, burning micturition, abdominal distension, or constipation.

On examination, vitals recorded were pulse 120 beats min, temperature 101°F, respiratory rate 22/min, and blood pressure 124/76 mmHg. General physical examination did not reveal pallor, scleral icterus, or palpable lymphadenopathy. Abdominal examination showed localized tenderness in the left hypochondrium, without guarding, rigidity, organomegaly, or signs of peritonitis. Respiratory, cardiovascular, and nervous system examinations were unremarkable. In the absence of definitive system-specific localizing signs, the patient was provisionally evaluated as a case of acute febrile illness/undifferentiated fever.

**Figure 1:**
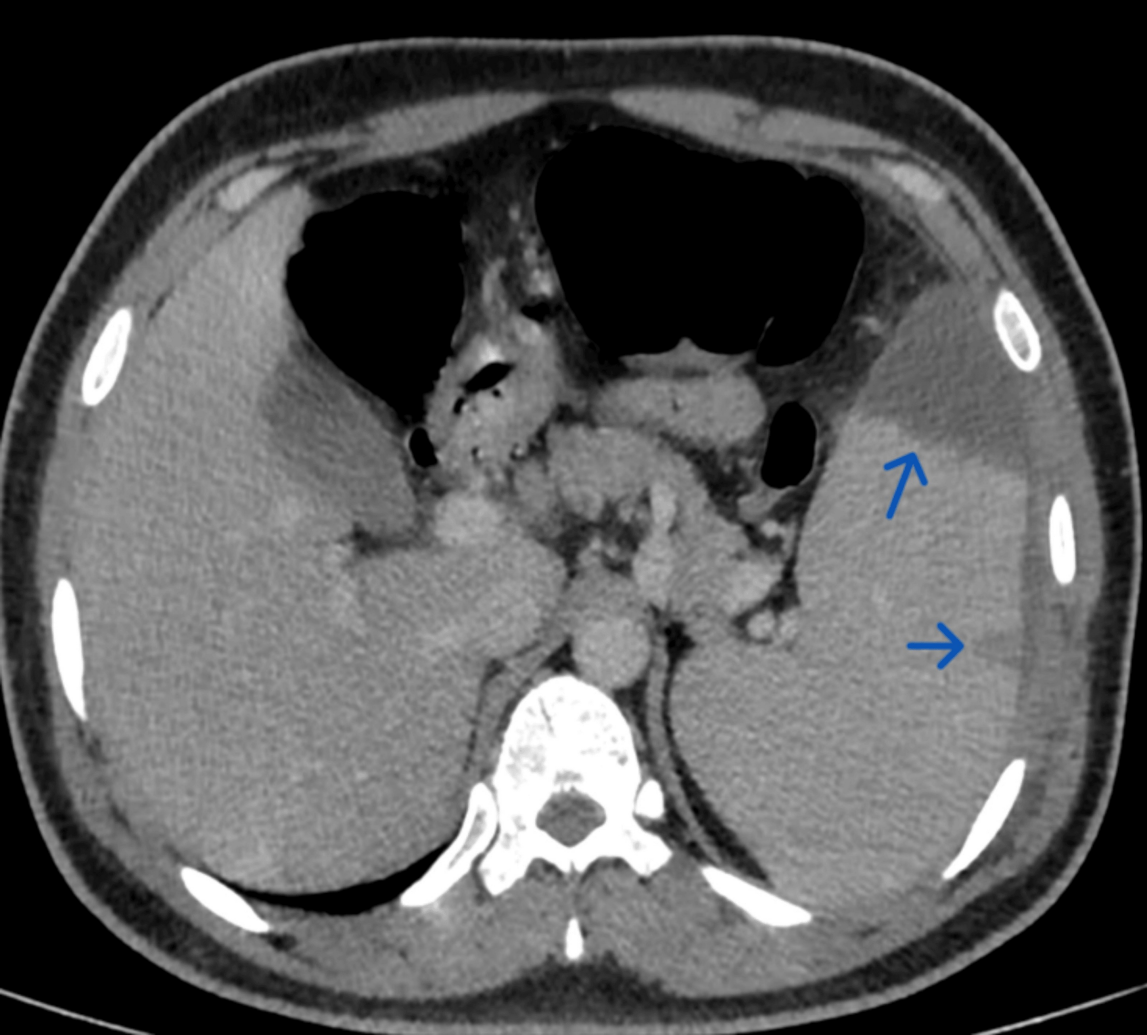
Contrast-enhanced computed tomography scan of the abdomen, axial section shows multiple wedge-shaped hypodense non-enhancing areas, suggestive of splenic infarcts (blue arrows)

**Figure 2:**
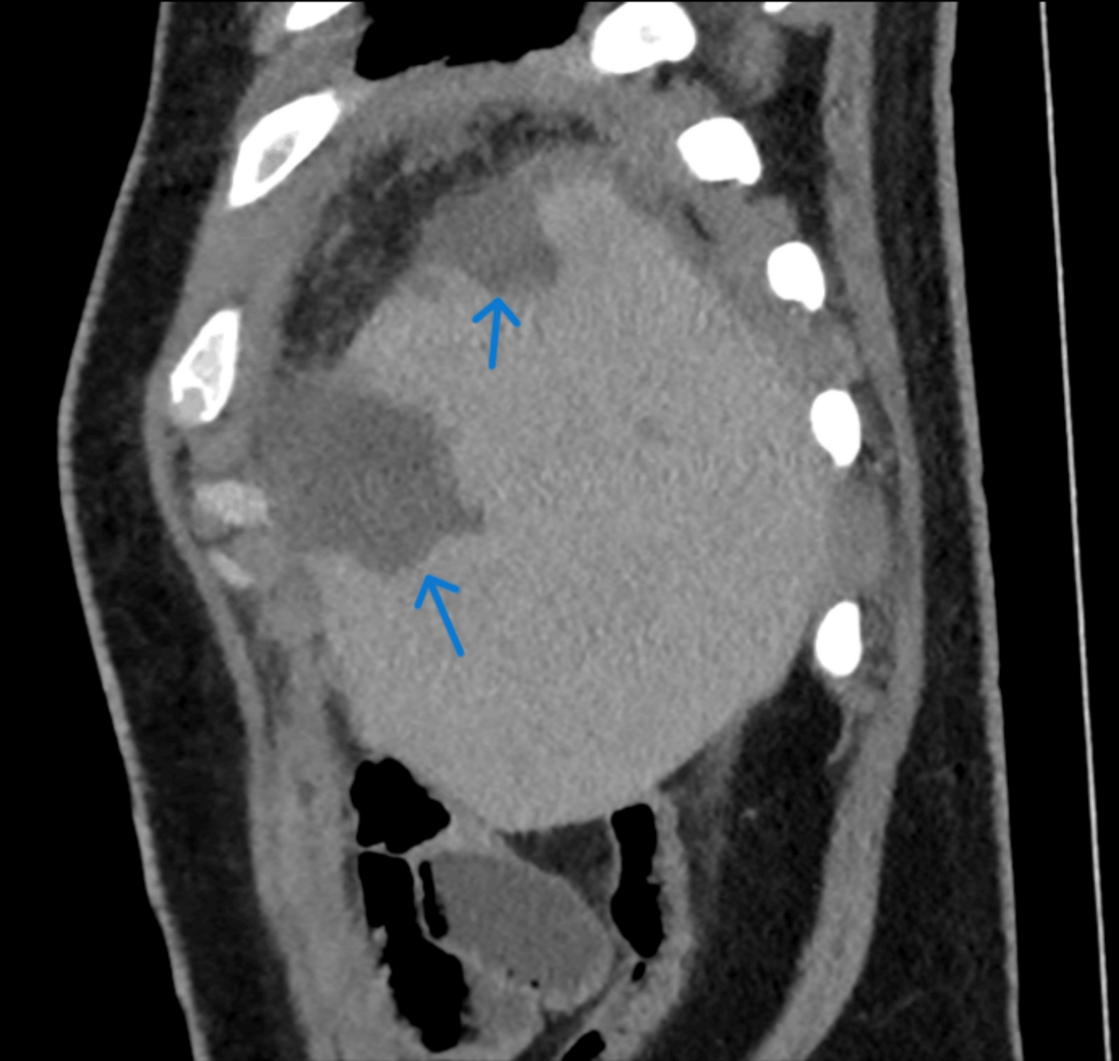
Sagittal reformatted CECT section shows enlarged spleen with peripherally based pyramidal-shaped infarcts in the splenic parenchyma (blue arrows) CECT: Contrast-enhanced computed tomography

Routine blood investigations (Table 1) revealed mild leukocytosis, deranged liver function tests, and raised C-reactive protein. Given localized pain, an ultrasound abdomen was performed, which showed splenomegaly with two peripheral hypoechoic wedge-shaped areas in the spleen, demonstrating the “bright band sign” suggestive of an infarct. This was followed by a contrast-enhanced CT scan of the abdomen, which confirmed hypodense, non-enhancing, wedge-shaped regions in the periphery of the splenic parenchyma, consistent with splenic infarcts (Figures 1, 2).

**Table 1:** Routine blood investigations RBC: Red blood cell count, TLC: Total leukocyte count, PT: Prothrombin time, INR: International Normalized Ratio, AST: Aspartate transaminase, ALT: Alanine transaminase, ALP: Alkaline phosphatase, CRP: C-reactive protein, LDH: Lactate dehydrogenase, DLC: Differential leukocyte count, ESR: Erythrocyte sedimentation rate

Parameters	On admission	On discharge	Reference range
Hemoglobin (g/L)	12.6	12.2	M – 13-16 g/L F – 12-15 g/L
RBC count (X 10^12^/L)	3.36	4.40	M - 4.5-5.5 X10^12 ^/L F - 3.8-4.8 X10^12^/L
TLC (X 10^9^ / L)	9.5	8.56	4-10 X10^9^/L
Neutrophils (%)	76		40-80%
Lymphocytes (%)	17		20-40%
Monocytes (%)	6		2-10%
Eosinophils (%)	1		1-6%
Platelet count (X 10^/9 ^/ L)	196	460	150-450 X10^9^/L
ESR (mm/hr)	40		M- 0-10 mm 1^st^ hr F- 0-10 mm 1^st^ hr
PT (sec)	14	13	10-14 sec
INR	1.02	1.0	0.8-1.2
Urea (mg /dl)	40	18	15-45 mg/dl
Creatinine (mg/dl)	1.1	0.9	0.8-1.8 mg/dl
Total bilirubin (mg/dl)	1.8	1.1	0.2-1 mg/dl
Conjugated bilirubin (mg/dl)	0.6	0.6	0.25 mg/dl
ALP (IU/L)	78	68	M - 40-130 IU/L F - 35-105 IU/L
AST (IU/L)	85	25	5-40 IU/L
ALT (IU/L)	97	20	5-35 IU/L
CRP (mg/L)	392	88	< 3 mg/L
LDH (U/L)	430		140-280 U/L
Amylase (IU/L)	22		22-80 IU/L
Lipase (IU/L)	19		13-60 IU/L

Given the short duration of symptoms, infective causes for splenic infarcts were initially considered. Blood cultures and a transthoracic echocardiogram were obtained early in the evaluation, showing no vegetations and a preserved cardiac function. Malaria antigen tests and peripheral blood film were negative. Serology for Brucella IgM, Hepatitis B, C, and HIV yielded negative results. Testing for viral etiologies such as Epstein-Barr virus and Cytomegalovirus (CMV) was deferred due to financial reasons. A Mantoux examination showed no induration and a normal chest roentgenogram virtually ruled out tuberculosis. A workup for hypercoagulable states (Protein C and Protein S activity) was also negative. Hemoglobin electrophoresis did not show any hemoglobin variants.

Blood culture grew non-lactose fermenting colonies, and the isolate was confirmed to be *Salmonella Paratyphi A* by serotyping, which was sensitive to ceftriaxone, azithromycin, and ciprofloxacin. Simultaneously, a Widal test revealed a Salmonella AH antigen titer of 1:320. The diagnosis was further supported by an ELISA dot assay, which showed a positive IgM result for *Salmonella Paratyphi A*. The patient was initially managed with intravenous fluids, antipyretics, and analgesics. Upon microbiological diagnosis, intravenous ceftriaxone was initiated for a duration of two weeks. The patient responded well to therapy, becoming afebrile by the third day of hospitalization with gradual resolution of symptoms and progressive improvement in liver function tests. After completing the course of antibiotics, the patient was discharged in stable condition. At the two-week follow-up, the patient was asymptomatic, and a repeat Widal test after four weeks from initial testing showed a four-fold fall in titer to 1:40.

## Discussion

Splenic infarction is most often seen with hematological malignancies such as leukemia and lymphoma, or with thromboembolic conditions such as infective endocarditis. Other causes include autoimmune or vasculitic disorders, sickle cell disease, and hypercoagulable states. Less commonly, infections such as *Staphylococcal aureus*, *Streptococcus* species, or *Escherichia coli* bacteremia, malaria, tuberculosis, CMV, Epstein-Barr virus, and human immunodeficiency viruses are implicated. Rarely, visceral leishmaniasis and fungal infections (e.g., Candida, Aspergillus, Histoplasma) may also lead to splenic infarction [6].

Existing literature has sparse data on the spectrum of splenic involvement secondary to *Salmonella* infection, which ranges from splenomegaly and splenic abscess to splenic thrombosis, infarction, and rupture. A study by Allal et al. reported an incidence of 2% (eight cases) of splenic abscess among 400 patients with typhoid fever [7]. Similarly, Im et al., in their analysis of over 350 patients with splenic infarction, identified more than 100 cases attributable to infectious etiologies, of which two were due to *Salmonella typhi* [6]. Another Indian study by Saini et al. reported a rare case of acute abdomen secondary to concurrent splenic vein thrombosis and multiple splenic infarcts, attributable to underlying *Salmonella paratyphii *infection [8]. Overall, most studies describing splenic involvement in the context of *Salmonella* infection are limited to case reports and case series, with large cohorts being scarce.

The underlying pathogenesis of splenic infarction in *Salmonella* infection is not well understood; however, several mechanisms have been postulated. Infection-induced endothelial injury triggering the inflammatory cascade, hypercoagulability, excessive deposition of circulating immune complexes in the microcirculation, and a mismatch in the demand and blood supply of the spleen have been proposed [9].

Splenic infarction typically presents with vague abdominal pain and tenderness, sometimes localizing to the left upper quadrant, along with nausea, vomiting, and fever [10]. Non-specific symptoms often cause a delay in diagnosis. A high index of clinical suspicion along with imaging is therefore essential when evaluating patients in the setting of abdominal pain with acute onset of fever, especially in those belonging to endemic areas or with a history of travel to such areas.

Timely diagnosis is very crucial as it may further complicate into a life-threatening splenic rupture or hemorrhage. Ultrasound abdomen is the most convenient imaging modality with a sensitivity of around 50% in detecting acute infarcts. Also, ultrasonographic findings, being subtle and operator dependent, are subject to error. While adding color Doppler marginally improves sensitivity, it remains inferior to contrast-enhanced computed tomography (CECT) of the abdomen. CECT is considered the gold standard imaging modality, offering nearly 100 % sensitivity and specificity. It can be utilized across the acute, subacute, and chronic phases and is particularly valuable for identifying associated vascular complications like splenic thrombosis [11].

Treatment of *Salmonella *infection in this particular scenario primarily involves appropriate antibiotic therapy, with the first option being Ceftriaxone 50 to 75 mg/kg/day in two divided doses for 10-14 days and/ or azithromycin 20mg/kg/day for seven days for inpatients [12]. Splenic infarction is generally managed with supportive measures, including adequate hydration, antiemetics, and analgesics. Surgical intervention of splenic infarction may be required in persistently symptomatic patients unresponsive to conservative therapy or in those who develop complications such as hemorrhage or splenic abscess.

However, due to the limited number of cases reporting splenic involvement as a complication of *Salmonella* infection, certain lacunae exist regarding its management. Firstly, there is insufficient evidence to support the benefits of prolonged antibiotic therapy or the use of antithrombotic agents. Secondly, challenges remain in identifying patients at risk of progression to life-threatening complications such as splenic hemorrhage or rupture. These patients would generally exhibit persistent or worsening symptoms, a continuing fall in hemoglobin, or signs of hemodynamic compromise. Thirdly, consensus is lacking on the need and timing of follow-up imaging to monitor resolution or detect delayed complications. Lastly, the role of elective splenectomy in asymptomatic, clinically recovered patients with large or chronic infarcts remains unclear.

## Conclusions

This case report underscores the importance of physicians to maintain a high index of suspicion for splenic infarction in patients presenting with persistent left upper quadrant abdominal pain and fever. Such cases warrant thorough evaluation for infectious etiologies, as common infections, including Salmonella in endemic regions like India, can present with atypical manifestations. Splenic infarction is a rare complication of enteric fever, where timely imaging facilitates accurate diagnosis and appropriate management, thereby preventing adverse outcomes. Prompt antibiotic therapy and supportive care usually lead to favorable recovery. Further research is essential to address the knowledge gaps and to establish standardized guidelines for optimal patient care.
